# Elevated plasma 20S proteasome chymotrypsin-like activity is correlated with IL-8 levels and associated with an increased risk of death in glial brain tumor patients

**DOI:** 10.1371/journal.pone.0238406

**Published:** 2020-09-04

**Authors:** Olga Martyna Koper-Lenkiewicz, Joanna Kamińska, Joanna Reszeć, Violetta Dymicka-Piekarska, Halina Ostrowska, Maria Karpińska, Joanna Matowicka-Karna, Marzena Tylicka

**Affiliations:** 1 Department of Clinical Laboratory Diagnostics, Medical University of Białystok, Białystok, Poland; 2 Department of Medical Pathomorphology, Medical University of Bialystok, Białystok, Poland; 3 Department of Biology, Medical University of Białystok, Białystok, Poland; 4 Department of Biophysics, Medical University of Białystok, Białystok, Poland; Goethe University Hospital Frankfurt, GERMANY

## Abstract

**Introduction:**

In cancer treatment an attempt has been made to pharmacologically regulate the proteasome functions, thus the aim was to test whether 20S proteasome chymotrypsin-like (ChT-L) activity has a role in glial brain tumors. Furthermore, we analyzed the correlation between proteasome activity and IL-8, CCL2, NF-κB1 and NF-κB2 concentrations, which impact on brain tumors has already been indicated.

**Methods:**

Plasma 20S proteasome ChT-L activity was assayed using the fluorogenic peptide substrate Suc-Leu-Leu-Val-Tyr-AMC in the presence of SDS. IL-8, CCL2, NF-κB1 and NF-κB2 concentration was analyzed with the use of ELISA method. Immunohistochemistry for IDH1-R132H was done on 5-microns–thick formalin-fixed, paraffin-embedded tumor sections with the use of antibody specific for the mutant IDH1-R132H protein. Labelled streptavidin biotin kit was used as a detection system.

**Results:**

Brain tumor patients had statistically higher 20S proteasome ChT-L activity (0.649 U/mg) compared to non-tumoral individuals (0.430 U/mg). *IDH1* wild-type patients had statistically higher 20S proteasome ChT-L activity (1.025 U/mg) compared to *IDH1* mutants (0.549 U/mg). 20S proteasome ChT-L activity in brain tumor patients who died as the consequence of a tumor (0.649) in the following 2 years was statistically higher compared to brain tumor patients who lived (0.430 U/mg). In brain tumor patients the 20S proteasome ChT-L activity positively correlated with IL-8 concentration.

**Conclusions:**

Elevated 20S proteasome ChT-L activity was related to the increased risk of death in glial brain tumor patients. A positive correlation between 20S proteasome ChT-L activity and IL-8 concentration may indicate the molecular mechanisms regulating glial tumor biology. Thus research on proteasomes may be important and should be carried out to verify if this protein complexes may represent a potential therapeutic target to limit brain tumor invasion.

## Introduction

Gliomas account for about 30% of all brain and central nervous system (CNS) tumors [1]. The most common malignant primary brain tumor originating from glial cells and one of the most expensive cancers to treat is glioblastoma (GBM). Patients’ prognosis is very poor due to the resistance of the tumor to chemo- and radiotherapy, the aggressive cancer phenotype and because of angiogenesis induced by the significant elevation of the vascular-endothelial growth factor (VEGF) expression [2, 3]. Statistics indicate that median patient survival is 14 months from diagnosis [4, 5]. Therefore glioblastoma is still a therapeutic challenge as a highly infiltrative, proliferative, and resistant tumor. Regulation of the cell cycle and the induction of the apoptosis by proteasome modulation, mainly by inhibiting chymotrypsin-like activity seems to be very promising in controlling tumor progression [6]. Some studies suggested that proteasomal activity is important in the process of tumor cell proliferation and development of tumor drug resistance [7]. Proteasome are large multicatalytic proteinase complexes which are responsible for more than 80% of the intracellular protein degradation, including proteins crucial to cell cycle regulation, apoptosis and programmed cell death [8]. They are involved in the degradation of a range of endogeneous proteins associated with cancer, including transcription factors, cyclins, Bax, p53, p27 and inhibitor of NF-κB and therefore they may become a potential target for cancer therapy [9]. In physiological condition NF-κB is maintained in a nonactive form due to complexing with its inhibitor (IκB). Activation of this nuclear factor by the proteasomal degradation of IκB leads to activation of antiapoptotic genes, resulting in cancer cell proliferation [10]. It has been reported that nuclear factor kappa B is highly expressed in a variety of malignant tumors such as pancreatic cancer, breast cancer and melanoma. Several studies suggested that NF-κB signaling pathway activation may play an important role in the occurrence and development of glioma [10]. There is also some evidence that proteasome may be involved in the carcinomas p27 level reduction, which is associated with poor prognosis. Studies on colorectal and lung cancer have demonstrated that the decrease of this protein is a consequence of the increased proteasome degradation. In astrocytic tumors the level of p27 decreases with advancing anaplasia and in the case of glioblastoma it is almost absent [11].

The release of NF-κB mediates the expression of interleukin-8 (IL-8), one of the CXC chemokines, which plays multiple roles in cancer pathophysiology [12]. IL-8 regulates apoptosis, proliferation, and migration of glioma cells and enhances angiogenesis [12, 13]. It is also recognized as an inflammatory chemoattractant for glioblastoma cells [14, 15], and moreover is released by these cells in an autocrine fashion [15, 16]. Studies of Zhang et al [17] indicated a significant correlation of IL-8 expression with the clinicopathological grades of gliomas. Furthermore, high IL-8 expression correlated with poor outcomes for glioma patients [17]. Among factors increasing IL-8 expression in glioma patients we can distinguish: necrotic cells [13], bradykinin [12], specific protein-1 (SP-1) [18], lipopolysaccharide (LPS) [19], prostaglandin E2 (PGE2) [20], or non-toxic concentrations of cadmium [21]. Another chemokine engaged in primary brain tumor pathogenesis is chemoattractant protein 1 (CCL2/MCP-1), a high expression of which was found in glioblastoma, anaplastic astrocytoma, and World Health Organization (WHO) grade 2 fibrillary astrocytoma [22, 23]. CCL2 is responsible for the accumulation of the tumor associated macrophages (TAMs) within the tumor environment, which secrete IL-8 [23].

In the treatment of some cancers an attempt has been made to pharmacologically regulate proteasome function [6, 24], thus it would be interesting to test the hypothesis that increased proteasome activity also plays a role in the pathogenesis of glial brain tumors. Because available studies indicated that isocitrate dehydrogenase 1 (*IDH1)* mutants showed better prognosis compared to *IDH*-wildtype glioma patients at different WHO grades [25], therefore in the next step we analyzed proteasome activity depending on the mutation of the *IDH1* gene. Further, we tested the correlation between proteasome activity and IL-8, CCL2, NF-κB1 and NF-κB2 concentrations. Finally, to explore whether proteasome activity is related to a worse prognosis for patients, we analyzed the aforementioned parameters depending on patients’ survival in the following 2 years.

## Material and methods

### Subjects

The research was conducted in agreement with the Helsinki-II-declaration and was approved by the Bioethics Human Research Committee of the Medical University of Bialystok (Permission No. R-I-002/383/2015). Subjects from the study and the control group were recruited between July 2015 and January 2018 at the Department of Neurosurgery of the Medical University of Bialystok. Samples were analyzed at the Department of Clinical Laboratory Diagnostics, Department of Biophysics and Department of Medical Pathomorphology of the Medical University of Bialystok.

The study group included 33 patients with a previously untreated primary brain tumor (22 males, 11 females; median age 59 years, range 39–73 years). All patients in the group were directed to surgical resection of the tumor. The inclusion criterion was the histopathological examination result indicating an astrocytic brain tumor diagnosis. Exclusion criteria included: a brain tumor remission in the patient’s medical history, neurodegenerative conditions like multiple sclerosis, neuroinfection, surgery or major trauma in the previous months.

The control group was composed of 10 non-tumoral subjects (3 males/7 females; median age 66 years, range 25–78 years) suffering from trigeminal neuralgia due to anatomical conflict between the trigeminal nerve and a cerebellar artery. All patients belonging to this group revealed refractory to conservative treatment and were qualified for posterior fossa craniotomy and microvascular decompression. Patients with any cancer in their medical history were excluded from the study. The remaining exclusion criteria from the control group were exactly the same as observed in the study group.

If one met the inclusion criteria, and gave an informed written consent to participate in the study, a sample was collected. Subjects included to the study were selected randomly, as not all of patients meting the criteria gave their permission for sample collection. Thus studied subjects can be considered as a probability sample, which could be considered representative of a larger population.

Before surgery all patients had fasting basic laboratory tests carried out between 6:00 AM and 7:00 AM. Brain tumor group was age-matched to the control group (p>0.05). Table 1 presents demographical data and routine laboratory test results of brain tumor patients versus non-tumoral individuals (Table 1). Table 2 presents demographical data, histopathological examination results and WHO grading of particular brain tumor patients (Table 2).

**Table 1:** Demographical data and routine laboratory tests results of brain tumor patients versus non-tumoral individuals. Differences were considered significant with the value of p<0.05.

	Brain tumor N = 33	Non-tumoral N = 10	p-value
**Age (years)**	**59** (48–67)	**66** (47–68)	0.561
**Gender: Male/Female**	**22/11**	**3/7**	-
**WBC [x10**^**3**^**/μl]**	**12.31** (8.31–17.90)	**6.02** (4.71–7.49)	***0*.*000***
**Na**^**+**^ **[mmol/l]**	**138** (136–140)	**140** (136–140)	0.682
**K**^**+**^ **[mmol/l]**	**4.6** (4.3–4.8)	**4.5** (4.2–4.7)	0.542
**Glucose [mg/dl]**	**114**(85–153)	**89** (86–103)	0.133
**Urea [mg/dl]**	**47** (38–58)	**30** (20–49)	***0*.*024***
**Creatinine [mg/dl]**	**0.78** (0.76–0.89)	**0.78** (0.69–0.83)	0.419
**eGFR [ml/min/1.73 m**^**2**^**]**	**104** (79–111)	**95** (77–104)	0.561

WBC—white blood cells count, eGFR—estimated glomerular filtration rate Conversion factor conventional to SI unit: WBC [10^9^/l]—1.0, creatinine [μmol/l]—88.402, eGFR [ml/s]—0.0167.

**Table 2:** Demographical data, histopathological examination results and WHO grading of particular brain tumor patients.

No.	Histopathological examination	Sex	Age	WHO	GFAP	p53	Ki-67	EGFR	IDH1
**1**	Diffuse astrocytoma	M	39	2	(+)	(+)	(+) in about 2% cells	(-)	(-)
**2**	Glioblastoma	M	45	4	(+)	(+) in about 10% cells	(+)in about 30% cells	(++)	(-)
**3**	Glioblastoma	F	67	4	(+)	(+) in about 20% cells	(+) in about 30% cells	(++)	(-)
**4**	Anaplastic astrocytoma	M	60	4	(+)	(+) in the single cells	(+) in about 20% cells	(+)	(-)
**5**	Diffuse astrocytoma	F	70	2	(+)	(+) in the single cells	(+) in about 1% cells	(-)	(-)
**6**	Glioblastoma	M	41	4	(+)	(+)	(+) in about 60% cells	(+) in parts of neoplastic tissue	(+)
**7**	Glioblastoma	F	73	4	(+)	(+)	(+) in about 20% cells	(+)	(+)
**8**	Glioblastoma	F	72	4	(+)	(+)	(+) in about 40% cells	(+)	(+)
**9**	Gliosarcoma	M	58	4	(+)	(+)	(+) in about 30% cells	(++)	(+)
**10**	Glioblastoma	M	55	4	(+)	(+)	(+) in about 30% cells	(+)	(+) focally
**11**	Anaplastic glioma	M	57	3	(+)	(+)	(+) in about 20% cells	(+)	(-)
**12**	Glioblastoma	F	44	4	(+)	(+) in about 30% cells	(+) in about 20% cells	(+)	(-)
**13**	Glioblastoma	M	57	4	(+)	(+)	(+) in about 40% cells	(+)	(+) in the single cells
**14**	Glioblastoma	F	62	4	(+)	(+) in about 30% cells	(+) in about 80% cells	(-)	(-)
**15**	Pilocytic astrocytoma	M	42	1	(+)	(+) in the single cells	(+) in 2% cells	(-)	(-)
**16**	Glioma	F	65	4	(+)	(+) in 40% cells	(+) in 20% cells	(+)	(-)
**17**	Glioblastoma	M	59	4	(+)	(+) in 20% cells	(+) in 30% cells	(++)	(-)
**18**	Glioblastoma	F	51	4	(+)	(+) in about 10% cells	(+) in about 10% cells	(+)	(+)
**19**	Glioblastoma	M	72	4	(+)	(+) in about 10% cells	(+) in about 10% cells	(+)	(-)
**20**	Anaplastic astrocytoma	F	40	3	(+)	(+)	(+) in about 10–15% cells	(+)	(-)
**21**	Glioblastoma	M	44	4	(+)	(+) in about 20% cells	(+) in about 30% cells	(-)	(+)
**22**	Glioblastoma	M	43	4	(+)	(+) in about 30% cells	(+) in about 50% cells	(-)	(+)
**23**	Glioblastoma	F	59	4	(+)	(+) in about 30% cells	(+) in about 40% cells	(+)	(-)
**24**	Glioblastoma	M	69	4	(+)	(+) in about 80% cells	(+) in about 30% cells	(+)	(+)
**25**	Glioblastoma	M	67	4	(+)	(+) in about 10% cells	(+) in about 30% cells	(+)	(-)
**26**	Glioblastoma	M	73	4	(+)	(+) in about 1% cells	(+) in about 60% cells	(+)	(-)
**27**	Glioblastoma	M	63	4	(+)	(+) in about 30% cells	(+) in about 30% cells	(+)	(+)
**28**	Glioblastoma	M	66	4	(+)	(+) in about 30% cells	(+) in about 20% cells	(+)	(+)
**29**	Glioblastoma	M	48	4	(+)	(+) in about 50% cells	(+) in about 40% cells	(+)	(+)
**30**	Glioblastoma	M	69	4	(+)	(+) in about 80% cells	(+) in about 20% cells	(+)	(-)
**31**	Glioblastoma	F	59	4	(+)	(+) in about 60% cells	(+) in about 20% cells	(-)	(-)
**32**	Glioblastoma	M	59	4	(+)	(+) in about 40% cells	(+) in about 30% cells	(+)	(-)
**33**	Glioblastoma	M	58	4	(+)	(+) in about 30% cells	(+) in about 10% cells	(+)	(+)

WHO—World Health Organization, GFAP—glial fibrillary acidic protein, p53—tumor protein p53, Ki-67—Ki-67 protein, EGFR—epidermal growth factor receptor, IDH1—isocitrate dehydrogenase 1, M—male, F—female.

### Sample collection and storage

Blood samples collected in tubes without anticoagulant (S-Monovette^®^, Sarstedt) and in EDTA-K3 tubes (S-Monovette^®^, Sarstedt) were centrifuged for 20 minutes at 1000 x g. The obtained serum and plasma supernatants were stored at -80 degrees Celsius until further analysis.

### The proteasome 20S chymotrypsin-like activity evaluation

Plasma proteasome chymotrypsin-like activity was assayed using the fluorogenic peptide substrate Suc-Leu-Leu-Val-Tyr-AMC in the presence of sodium dodecyl sulfate (SDS)–selective enzyme activator, as described by Ma et al [26] and Tylicka et al [27, 28]. 20S proteasome in plasma has been activated by the addition of sodium dodecyl sulfate because they cannot efficiently degrade peptides or proteins which are not highly denatured. Activation with SDS facilitates the infiltration of the protein substrate into the proteasome channel.

The activation of plasma samples was carried out with 5μL of 10% SDS for 15 min at room temperature. The concentration of sodium dodecyl sulfate in the plasma activation phase was 1%. Subsequently 10 μL of samples were carried to reaction wells containing 30 μL of an assay buffer (0.05% SDS in 100 mM Tris/HCl, pH = 7.5) and 10 μL of the fluorogenic peptide AMC substrate, so that the total volume of the reaction mixture was 50 μL and concentration of SDS was 0.03% (concentration needed to maximal activation of the 20S proteasome). The chymotryptic-like activity of the proteasome cleaves the Suc-Leu-Leu-Val-Tyr-AMC leading to the release of free AMC (7-amino-4-methylcoumarin) the quantity of which was measured using a fluorescence microplate reader FLUOstar OPTIMA (BMG Labtech, Germany). The reaction mixture was incubated at 37°C in a fluorescence microplate reader. Fluorescence was monitored at 3 min intervals during a 60-min incubation period at an excitation wavelength of 355 nm and an emission wavelength of 460 nm. The amount of AMC released from the substrate per minute was expressed as one unit of the 20S proteasome chymotrypsin-like activity (pmol/min = U). Specific activity was expressed in a unit by the amount of total protein (U/mg), the concentration of which, in plasma samples, was determined by the Bradford method, using the Bio-Rad assay reagent with bovine serum albumin as the standard. All assays were performed in triplicates.

**Fig 1:**
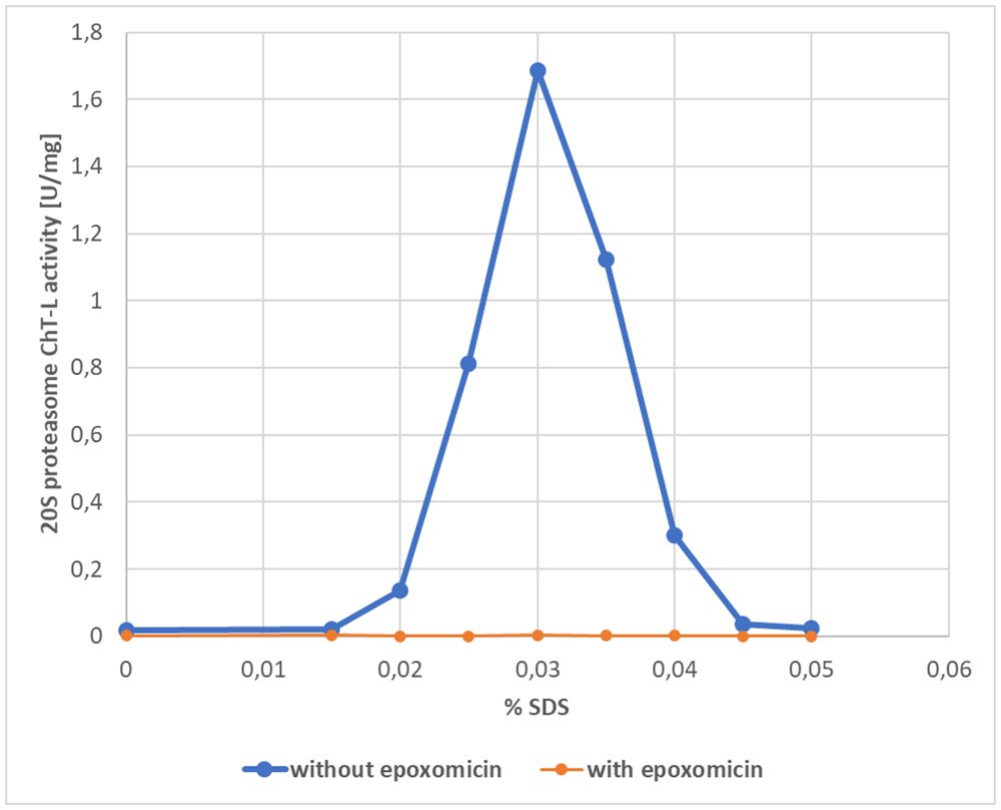
Sodium dodecyl sulfate (SDS) activation profile for Suc-Leu-Leu-Val-Tyr-AMC hydrolyzing activity in the absence or presence of the proteasome inhibitor epoxomicin in plasma of patient with brain tumor.

Based on our earlier research [29] plasma samples were pre-incubated with the selective proteasome inhibitor epoxomicin (1.0 μMol/L) for 15 min before the addition of a substrate to confirm the specificity of the assay. Verification, if the addition of SDS causes a real increase of proteasome catalytic activity, was established in our previous study during which plasma 20S proteasome activity with both the addition and subtraction of sodium dodecyl sulfate was measured [28]. The SDS activation profile for Suc-Leu-Leu-Val-Tyr-AMC hydrolyzing activity in the absence or presence of the proteasome inhibitor epoxomicin in the plasma of a brain tumor patient is presented in Fig 1.

### Serum IL-8 and CCL2 concentration analysis

IL-8 and CCL2 concentrations were analyzed with the use of an immunological ELISA (enzyme-linked immunosorbent assay) commercially available kit (R&D Systems Europe Ltd., Abingdon, England) in compliance with the manufacturer’s instructions. IL-8 concentration was measured by means of a Quantikine^®^ Human CXCL-8/IL-8 Immunoassay kit (Catalog number: D8000C). Serum was not diluted prior to analysis. The manufacturer of the assay kit referred to the intra-assay coefficient of variation (CV%) as 5.6% at IL-8 mean concentration of 168 ± 9.4 pg/mL. The detection range for the used CXCL-8/IL-8 ELISA kit is between 0.00–2000 pg/mL. CCL2 concentration was measured by means of a Quantikine^®^ Human CCL2/MCP-1 Immunoassay kit (Catalog number: DCP00). Serum samples were diluted 2-fold prior to analysis. The manufacturer of the assay kit referred to the intra-assay coefficient of variation (CV%) as 7.8% at CCL2 mean concentration of 76.7 ± 6.0 pg/mL. The detection range for the used CCL2/MCP-1 ELISA kit is between 0.00–2000 pg/mL.

### Plasma NF-κB1 and NF-κB2 concentration analysis

Plasma samples were not diluted prior to analysis. NF-κB1 (p105) concentration was measured using a Human NFKB1 kit (Catalog number orb563366) from Biorbyt Ltd., Cambridge, England. The detection range for the kit is between 0.0–20 ng/mL. The manufacturer of the assay kit referred to the intra-assay coefficient of variation (CV%) as 8%. NF-κB2 (p100) concentration was measured using a Human NFKB2 kit (Catalognumber: orb563844) from Biorbyt Ltd., Cambridge, England. The detection range for the kit is between 0.0–20 ng/mL. The manufacturer of the assay kit referred to the intra-assay CV% as 8%.

### IDH1 mutation detection

Immunohistochemistry for IDH1-R132H was done on 5-micron–thick formalin-fixed, paraffin-embedded tumor sections with the use of antibody specific for the mutant IDH1-R132H protein (H09, Dianova, dil 1:100). A labelled streptavidin biotin kit was used as a detection system (Agilent, Denmark)). Antigen retrieval was performed in citrate buffer (pH, 6.0) in a pTLink (Agilent).

Combined cytoplasmic and nuclear staining was interpreted as immunopositive. The 3-tiered semiquantitative system results were as follows: negative, if no tumor cell was immunopositive; partly positive (focal positivity), if there was an admixture of immunopositive and immunonegative tumor cells or areas of immunopositive and immunonegative tumor cells were adjacent to each other; complete positivity (diffuse positivity), if all the tumor cells were immunopositive.

### Statistical data analysis

Statistical analysis was performed using the STATISTICA PL release 12.5 Program. All results are presented as median with 25^th^ and 75^th^ percentiles (interquartiles, IQs). The data was tested for normality using the Shapiro-Wilk test. As the tested parameters did not follow the normal distribution, to compare two independent study groups the non-parametrical Mann-Whitney U test (two-sided nature) was used. Correlation coefficients analysis was obtained by applying Spearman’s rank method. A Receiver operator characteristic (ROC) curve was generated to calculate the area under the ROC curve (AUC). The Youden index, which is a function of sensitivity and specificity, was estimated to indicate an optimal cut-off value. Within the whole brain tumor group we also performed Kaplan-Meier survival analysis. Differences between variables were considered significant with the value of two-tailed p<0.05.

## Results

The 20S proteasome chymotrypsin-like (ChT-L) activity, NF-κB1, NF-κB2, IL-8 and CCL2 concentration were analyzed retrospectively. The 20S proteasome ChT-L activity, NF-κB1 and NF-κB2 concentration were analyzed in 31 brain tumor samples and 10 non-tumoral individuals (in 2 brain tumor cases we did not have preserved plasma samples). IL-8 concentration was analyzed in 33 brain tumor patients and 10 control subjects. CCL2 concentration was analyzed in 32 brain tumor patients and 10 control individuals (in 1 case we did not have a sufficient serum volume to conduct analysis). If a case had missing data for any of the variables, it was simply excluded from the analysis. It is usually the default in statistical packages [30].

### The proteasome 20S ChT-L activity results

**Fig 2:**
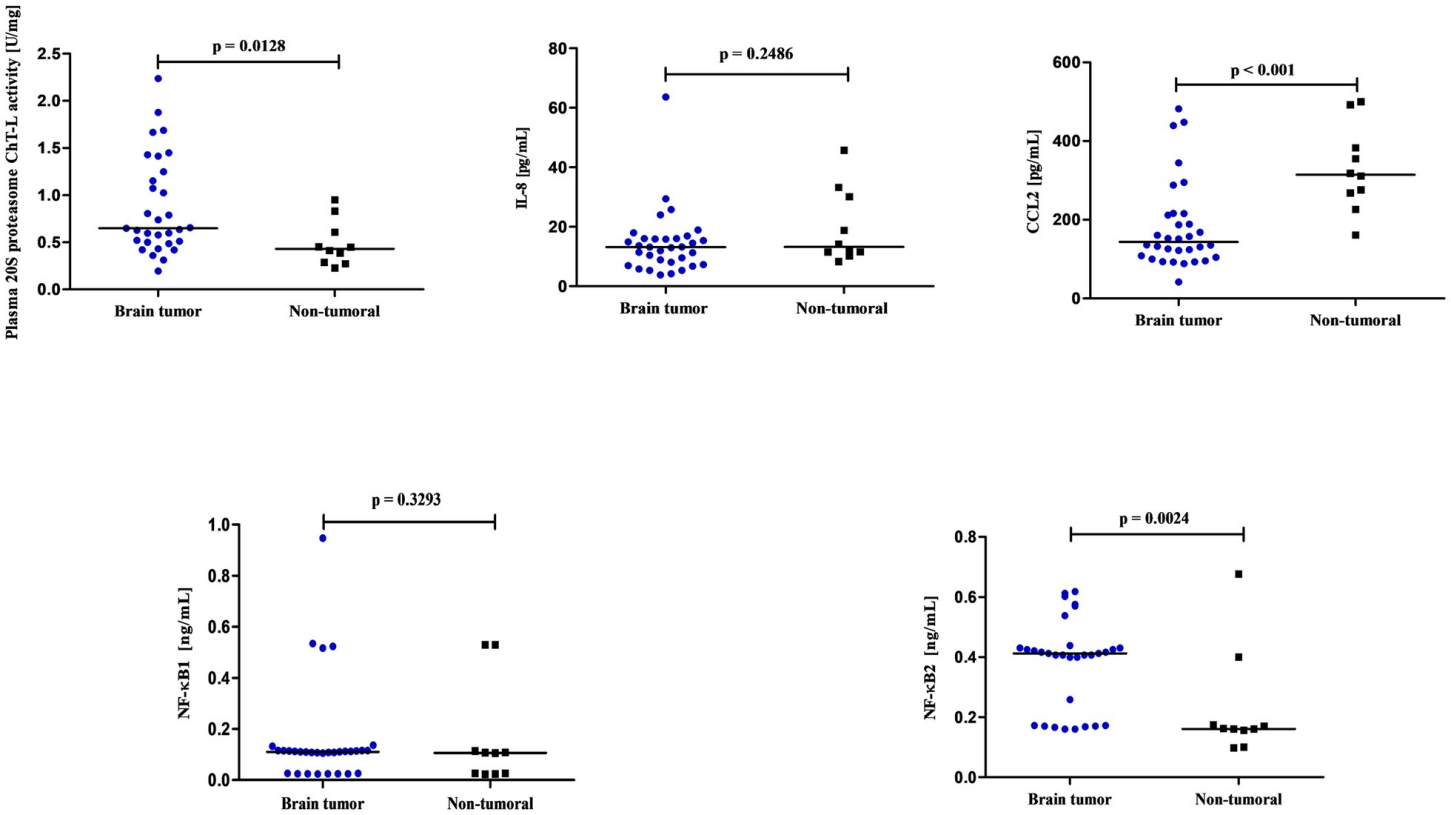
The 20S proteasome chymotrypsin-like (ChT-L) activity, IL-8, CCL2, NF-κB1 and NF-κB2 concentrations in brain tumor patients compared to non-tumoral individuals.

**Fig 3:**
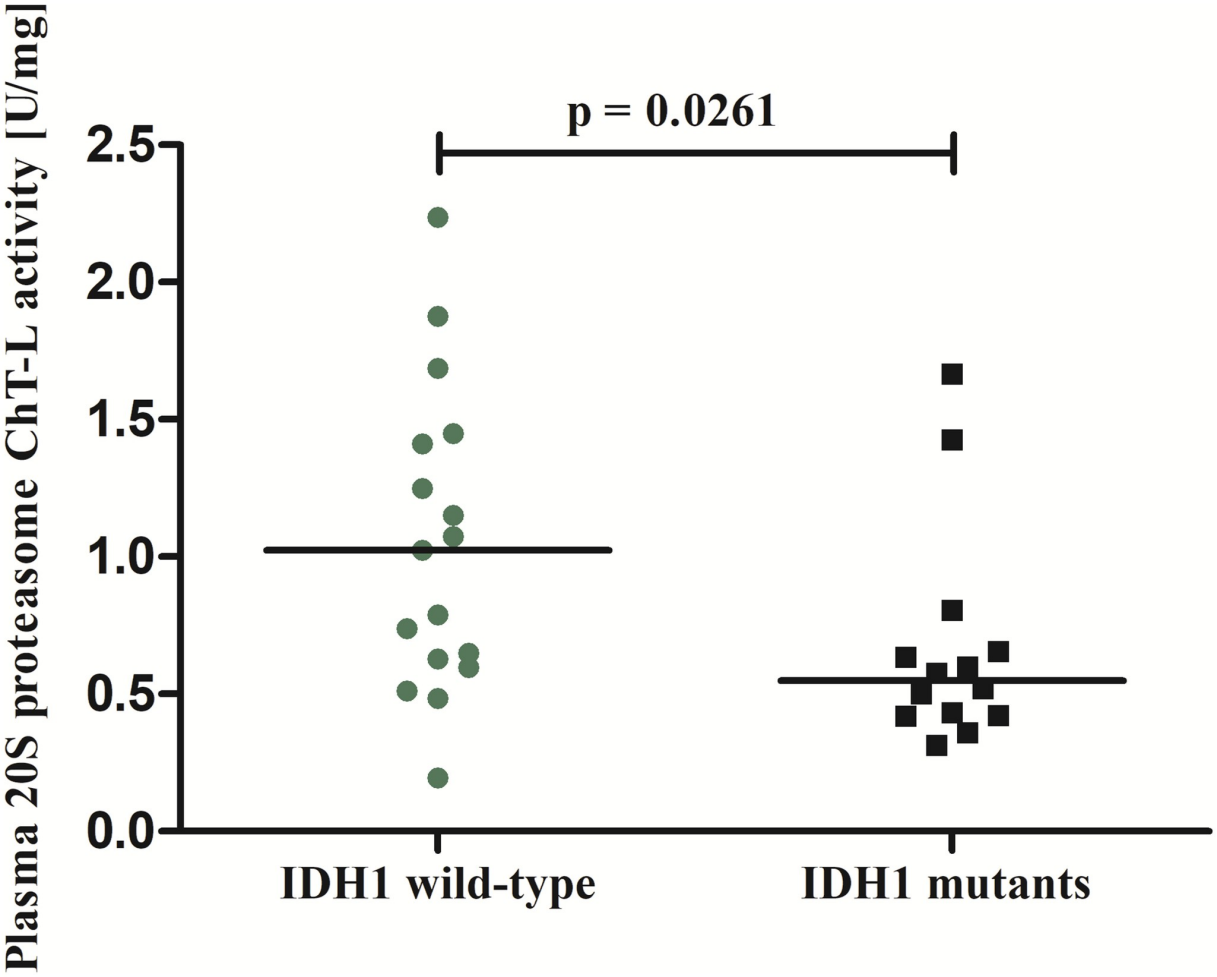
The 20S proteasome chymotrypsin-like (ChT-L) activity in brain tumor patients depending on the *IDH1* mutation.

**Fig 4:**
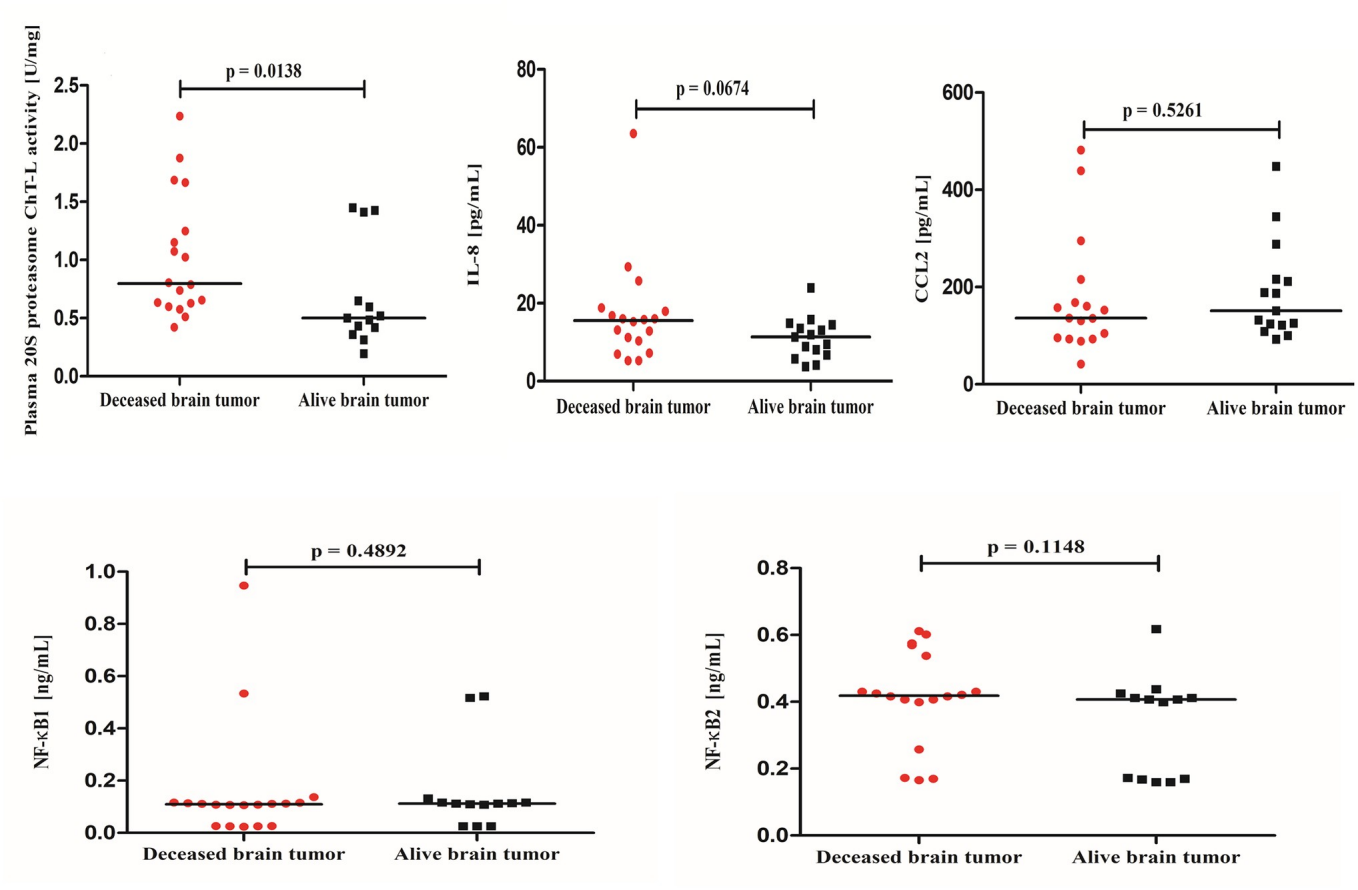
The 20S proteasome chymotrypsin-like (ChT-L) activity, IL-8, CCL2, NF-κB1 and NF-κB2 concentrations in brain tumor patients who died compared to brain tumor patients who survived the following two years.

Brain tumor patients had statistically higher 20S proteasome ChT-L activity compared to non-tumoral individuals (p = 0.0128) (Fig 2). To analyze how much the low grade tumors (1 and 2 WHO grade) influence the entire data including the 20S proteasome ChT-L activity, in the next step we compared only WHO grade 3–4 brain tumor patients to the control subjects. The proteasome 20S ChT-L activity in WHO grade 3–4 astrocytic brain tumor patients (N = 29) was also statistically higher (0.635 U/mg) compared to the control group (0.430 U/mg) (p = 0.0179). Analysis of the 20S proteasome ChT-L activity in brain tumor patients depending on the *IDH1* mutation found that *IDH1* wild-type individuals had statistically higher proteasome activity compared to *IDH1* mutants (p = 0.0261) (Fig 3). Elevation in 20S proteasome ChT-L activity in the brain tumor group was also related to an increased risk of death for these patients. The median value of this parameter in brain tumor patients who died as a consequence of a tumor in the following 2 years was statistically higher compared to brain tumor patients who lived (p = 0.0138) (Fig 4).

### IL-8 and CCL2 concentration results

Serum IL-8 concentration was higher in brain tumor patients as compared to the control group, but the difference was not significant (p = 0.2486) (Fig 2). We also did not observe a significant difference between IL-8 concentrations in brain tumor patients who died as a consequence of disease in the following 2 years compared to brain tumor patients who survived (p = 0.0674) (Fig 4).

Serum CCL2 concentration was statistically lower in brain tumor patients compared to the non-tumoral group (p<0.001) (Fig 2). Similarly as with the IL-8 results, there was no association between CCL2 concentration and an increased risk of death in brain tumor patients in the following 2 years, as chemokine levels did not differ between patients who had died compared to patients who had lived (p = 0.5261) (Fig 4).

### NF-κB1 and NF-κB1 concentration results

Plasma NF-κB1 concentration in brain tumor patients was similar to the control group (p = 0.3293). We did not observe a difference between NF-κB1 concentration in brain tumor patients who died compared to brain tumor patients who survived the following two years (p = 0.4892). Plasma NF-κB2 concentration in brain tumor patients was significantly higher compared to the control group (p = 0.0024). Similarly as with NF-κB1, we also did not observe a difference between the NF-κB2 concentration in brain tumor patients who died compared to brain tumor patients who survived the following 2 years (p = 0.1148) (Figs 2 and 4).

### Correlation coefficient analysis results

**Fig 5:**
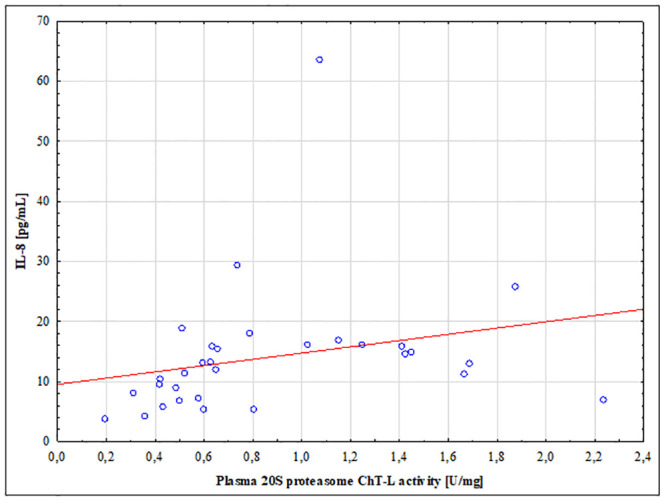
Correlation coefficient results between the 20S proteasome chymotrypsin-like (ChT-L) activity and IL-8 concentration in brain tumor patients.

The 20S proteasome ChT-L activity was correlated with IL-8, CCL2, NF-κB1 as well as NF-κB2 concentration. In brain tumor patients the 20S proteasome ChT-L activity showed a positive correlation with only IL-8 concentration (r = 0.508; p = 0.0031) (Fig 5); we did not observe a relationship between the 20S proteasome ChT-L activity and CCL2, NF-κB1 as well as the NF-κB2 concentration (p>0.05). In the control group we did not observe any correlation between the proteasome ChT-L activity and IL-8, CCL2, NF-κB1 as well as the NF-κB2 concentration (p>0.05).

### Receiver operated characteristic (ROC) curve analysis

**Fig 6:**
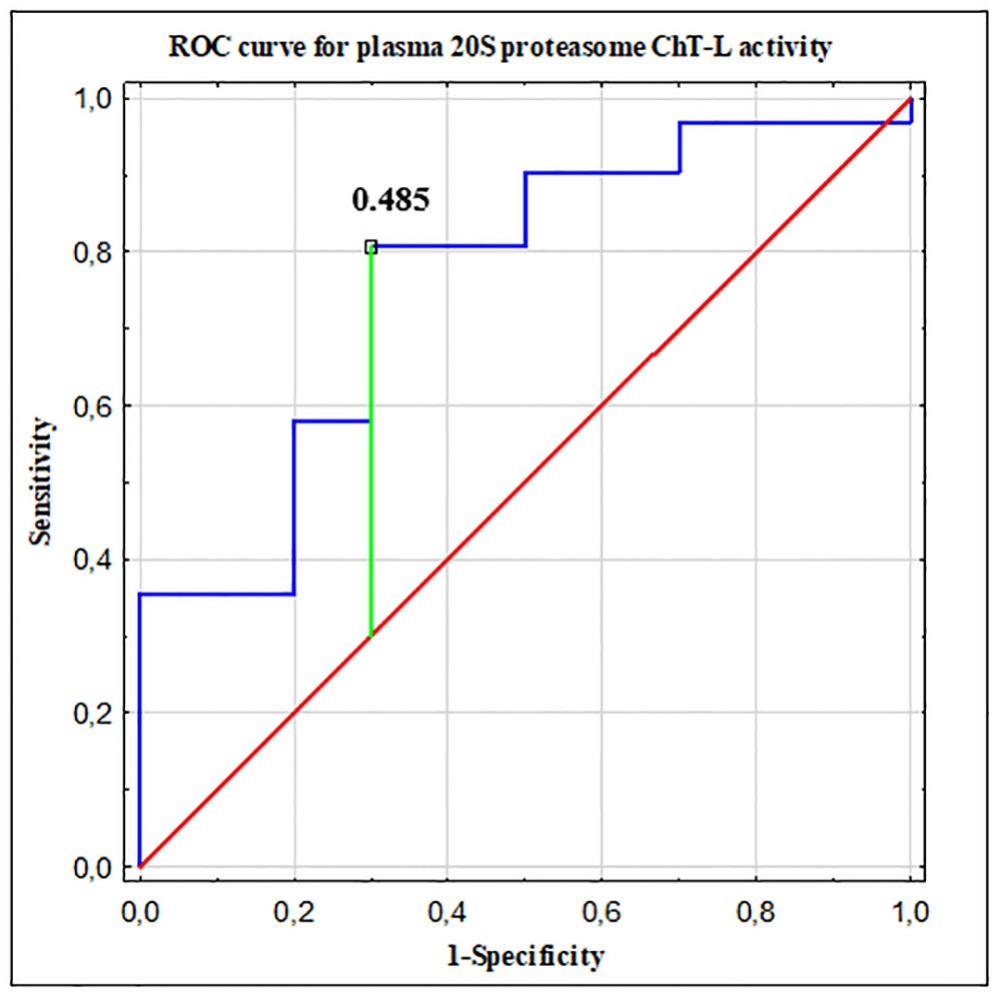
Receiver operated characteristic (ROC) curve analysis in differentiating brain tumor patients from non-tumoral individuals. The area under the ROC curve (AUC) for the 20S proteasome ChT-L activity equal to 0.761, the optimal cut-off point equal to 0.485 U/mg.

To indicate the optimal threshold value useful in differentiating brain tumor patients from non-tumoral individuals we conducted the ROC curve analysis. The optimal cut-off point for the 20S proteasome ChT-L activity was 0.485 U/mg. The area under the ROC curve (AUC) for the 20S proteasome ChT-L activity was statistically higher than the value of 0.5 **(**Fig 6, Table 3**)**.

**Table 3:** Diagnostic utility of the 20S proteasome ChT-L activity evaluation to discriminate primary brain tumor patients from non-tumoral individuals.

	AUC ± SE	p-value	Youden Index	Cut-off	Sensitivity	Specificity	Diagnostic accuracy
20S proteasome ChT-L activity	0.761 ± 0.087	0.0026	0.51	0.485 U/mg	81%	70%	78%

ChT-L—chymotrypsin-like; AUC—area under the ROC curve; SE—standard error; Cut-off—optimal cut-off based on the highest Youden Index.

### Kaplan-Meier survival analysis

Survival status for the studied patients was updated in February 2020. The median survival-time was 435 days (range 3–730 days). During this period 18 (55%) astrocytic brain tumor patients had died.

**Fig 7:**
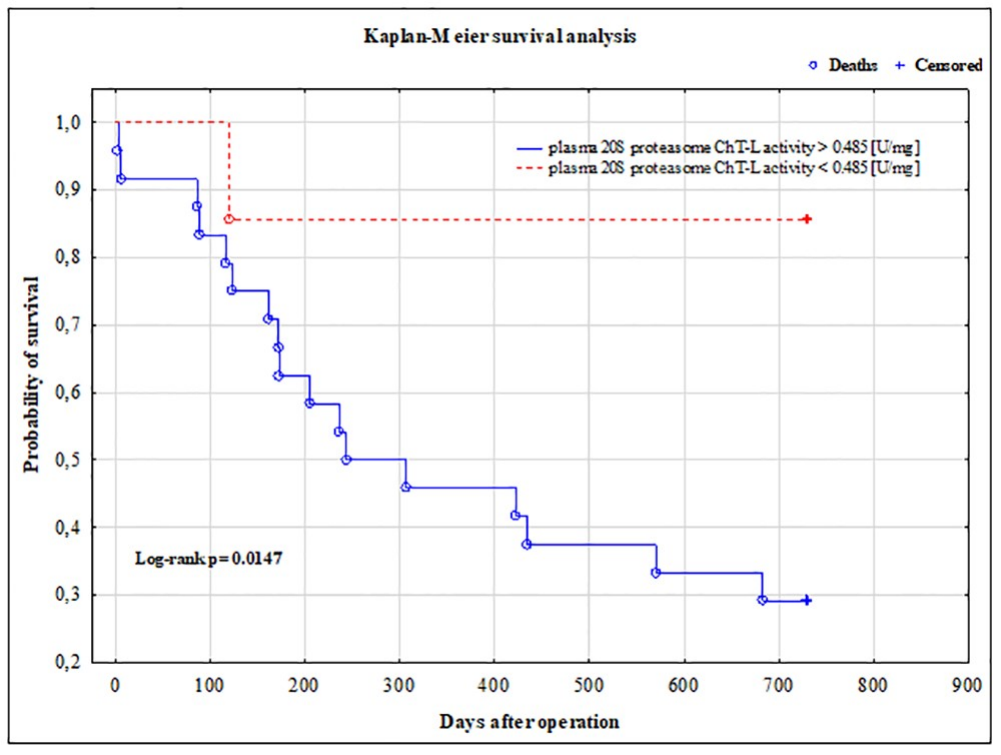
Kaplan-Meier survival analysis for brain tumors patients. Based on the optimal cut-off point for the 20S proteasome ChT-L activity useful in differentiating brain tumor patients from non-tumoral individuals tumoral subjects were divided into those with 20S proteasome ChT-L activity <0.485 U/mg and those with 20S proteasome ChT-L activity ≥0.485 U/mg.

Based on the optimal cut-off point for the 20S proteasome ChT-L activity useful in differentiating brain tumor patients from non-tumoral individuals we divided astrocytic brain tumor patients into those with 20S proteasome ChT-L activity <0.485 U/mg and those with 20S proteasome ChT-L activity ≥0.485 U/mg. The overall survival (OS) curves of these subgroups were significantly different (P = 0.0147) (Fig 7).

## Discussion

The proteasome activity significantly affects the course of major biological processes. Decreasing proteasome activity impairs the degradation of pathological proteins and causes their accumulation in the cell, while overactive proteasome activity causes deficiency of proteins necessary for the functioning of the body. Evaluation of proteasome activity, as a marker of the course of various diseases, may be useful in their diagnosis [24]. Attempts are currently being made to pharmacologically regulate the function of proteasome [24, 31–34], which indicates another potential practical application of proteasome activity analysis in the course of various diseases.

In the current study we found enhanced 20S proteasome chymotrypsin-like (ChT-L) activity in glial brain tumor patients, which moreover was related to an increased risk of death and worse clinical outcomes for these patients. Elevated 20S proteasome ChT-L activity in the glial brain tumor group compared to non-tumoral subjects may indicate a specific protection of cancer cells against apoptosis. Many cancers are connected with higher proteasome activity leading to degradation of proapoptotic proteins of the Bcl-2 family, negative cell cycle regulators (p53, p21Waf1/Cip1, p27Kip1) and IκB protein. Degradation of IκB leads to transcription factor NF-κB activation, which induces cancer cell survival and promotes inflammation [35].

The Mammalian NF-κB family encompasses five members: RelA/p65, c-Rel, RelB, NF-κB1 p50, and NF-κB2 p52 [36, 37]. They participate in various biological processes, including immune response, inflammation, cell growth, survival, and development. NF-κB1 and NF-κB2 are synthesized as precursor proteins, p105 and p100. Generally, NF-κB activation occurs by release from the IκB molecules or by cleavage of the inhibitory ankyrin repeat domains of NF-κB1 (p105) and NF-κB2 (p100). This is achieved by the proteasomal degradation of the IκB or by partial degradation of the precursors p105 and p100 [38]. In our research, both brain tumor patients as well as control subjects had relatively small plasma NF-κB1 (p105) and NF-κB2 (p100) concentration. On the one hand this could be related to anti-inflammatory treatment to which the study patients were subjected [39]. On the other hand the study of Moorthy et al [40] present that 20S proteasomes endoproteolytically cleave the fully synthesized p105 and selectively degrade leading to p50 generation in an ubiquitin-independent manner. The authors suggest that the basal degradation is mediated by the core 20S proteasomes which supports the notion that the 20S, but not the 26S, carries out p105 processing *in vivo*. Thus increased proteasome 20S ChT-L activity in patients with brain tumors compared to non-tumoral individuals may explain the low concentration of precursor proteins p105 in the current study.

On the other hand, the processing of p100 is tightly regulated by the non-canonical pathway of NF-κB activation and depends on both phosphorylation and ubiquitination [41] which can explain that NF-κB2 concentration was statistically higher in brain tumor patients compared to non-tumoral individuals. It may suggest, that in astrocytic brain tumor patients the non-canonical NF-κB pathway is activated, leading to the inducement of RelB:NF-κB2 heterodimer in the nucleus. In this pathway, activation of the NF-κB inducing kinase (NIK) after stimulation resulted in phosphorylation and a following proteasomal refinement of the precursor protein p100 into the mature NF-κB2 (p52) subunit in an IKKα/IKK1 dependent manner [42].

We also found that brain tumor *IDH1* wild-type individuals had statistically higher ChT-L proteasome activity compared to *IDH1* mutants. Isocitrate dehydrogenase 1 (IDH1) is an enzyme of the Krebs cycle that is the final pathway for the oxidation of proteins, fatty acids and carbohydrates. IDH1 catalyzes the conversion of isocitrate to alpha-ketoglutarate and plays a role in regulating the activity of the chain of subsequent oxidative phosphorylation, the final effect of which is the formation of ATP, GTP and precursors of significant chemical compounds [43]. *IDH1* mutation is observed in more than 80% of WHO II and WHO III grade malignant gliomas and secondary GBM, while they are very rare in primary GBM, ependymomas and hepatocellular astrocytoma [44]. The consequence of the occurrence of the *IDH1* mutation is a decrease in the enzyme activity and a decrease in the alpha-ketoglutarate concentration, which is a substrate for prolyl hydroxylases. These hydroxylases under normal oxygen concentration in the cells break down the alpha subunit of hypoxia inducible factor 1 (HIF1). In a situation of alpha-ketoglutarate deficiency, the cells behave as if they were hypoxic. The biological effect of the *IDH1* mutation therefore partially depends on HIF1 induction [45].

The survival rate for malignant glioma patients is still very poor, mostly due to resistance of glioma cells against applied therapy [46]. In the current study we found that enhanced proteasome ChT-L activity in the brain tumor group was significantly related to the poor prognosis in these patients. This is the reason why proteasome should represent a potential target for novel anti-brain tumor therapies and clearly indicates the usefulness of proteasome ChT-L activity evaluation.

Proteasome inhibitors encompass chemotherapeutic drugs with the ability to block NF-κB activity [32]. Alvarez-Castelao et al [47] show that 20S proteasomes can be directly involved in degradation of NF-κB inhibitors. Our research indicated that elevated 20S proteasome ChT-L activity may have an impact on NF-κB activation by degradation of NF-κB1 and NF-κB2 which in the form of homodimers serve as NF-κB inhibitors.

The proteasome activity inhibitor named bortezomib (MLN341, PS-341, LDP-341) is currently applied in clinical trials for cancer treatment, including brain tumors [31–34]. Unfortunately, it seems that bortezomib failed to inhibit the growth of gliomas *in vivo*, as phase I clinical trial of Phuphanich et al [32] did not indicate the efficacy of bortezomib in these patients. The failure of this therapy probably resulted from the limited penetration through the blood brain-barrier (BBB) [48], which is responsible for the selective transport of soluble and cellular factors between blood and the central nervous system [49]. Therefore in the future new proteasome inhibitors, enabling easier penetration of the BBB, will be developed. A novel proteasome inhibitor against gliomas could be SC68896 inducing cell cycle arrest and apoptosis in malignant cells and moreover sensitizing glioma cells to death ligands [46]. Therefore a practical aspect of our findings is the possibility of using the 20S proteasome ChT-L activity evaluation in routine clinical assessment of brain tumor patients’ response to applied proteasome inhibitor chemotherapy.

Increased proteasome activity leads to an elevation in the transcription factor NF-κB, that in turn causes IL-8 expression elevation [12] both in endothelial cells as well as in GBM cells [50]. As a consequence angiogenesis- and stem cell-related signaling pathways are activated. IL-8 promotes vascularization via growth factors modulation, mobilization of enzymes responsible for matrix remodeling, and moreover activation of tumorigenesis-associated pathways: STAT3 (Signal Transducer and Activator of Transcription 3), MAPK (Mitogen Activated Protein Kinase), and PI3K (phosphoinositide 3-kinase) [50]. In the present study we found a positive correlation between proteasome ChT-L activity and serum IL-8 concentration, which also indicates the direct relationship between altered proteasome activity and a worse outcome for brain tumor patients. Interestingly, we did not observe a relationship between the proteasome ChT-L activity and serum CCL2 concentration, which is another potential factor responsible for cancer progression [51, 52].

Data presented in the Cancer Genome Atlas and Ivy Glioblastoma Atlas Project showed that there is a negative correlation between IL-8 transcript expression and patient survival with GBM. IL-8 transcript expression moreover positively correlated with KLF4 (Kruppel-like factor 4), c-Myc oncogene, and HIF2α [53], which are molecular hallmarks of glioma-initiating cells (GICs). Studies of Hasan et al [53] in murine models found that IL-8 knockdown significantly decreased patient-derived xenograft (PDX) GBM tumor growth in vivo. The authors also observed that TMZ-induced IL-8 increases the trimethylation of histones H3K27 and H3K9, which indicates that IL-8-mediated signaling takes part in regulation of glioblastoma cells to stress caused by applied chemotherapy.

One limitation of the study could be the small number of studied patients. The number of observations is the basis for calculating the number of degrees of freedom for the analyzed test, and thus has an impact on whether the obtained result will be statistically significant or not. On the other hand, the larger the number, the smaller the difference between the examined groups turns out to be statistically significant [54]. In our study, despite the small number of cases, we obtained statistically significant differences. We also did not perform the sample size calculation, which could also be considered as a study limitation. However, it is usually done in big cooperative studies including cohorts. We only aimed to test the hypothesis whether the evaluation of proteasome ChT-L activity could be considered as an objective and quantifiable marker of glial brain tumors. Usually only the I-st type of statistical errors is considered in this type of “preliminary” study (error of false rejecting a null hypothesis) whereas the II-nd type (error of false admitting of an alternate hypothesis) is not taken into consideration. As the latter is usually bigger by a large margin, those studies should be undertaken to reach a real, final conclusions.

In the current study we measured the proteasome ChT-L activity, NF-κB1, NF-κB2, IL-8, and CCL2 in the blood only, which is another study limitation. Moreover, the p65/RelA concentration should also be measured. It would be also interesting if the 20S proteasome ChT-L activity correlates with these proteins at the cellular level. Thus further studies should be designed with the use of glioblastoma (GBM) tissue and GBM cell lines. Nevertheless this aspect deserves to be developed further.

Our findings indicate that elevated 20S proteasome ChT-L activity is related to an increased risk of death and worse clinical outcomes in glial brain tumor patients. A positive correlation between 20S proteasome ChT-L activity and serum IL-8 concentration may indicate the molecular mechanisms regulating glial tumor biology. Therefore 20S proteasome ChT-L activity control and regulation may represent a potential therapeutic target to limit brain tumor invasion. Elevated 20S proteasome ChT-L activity may affect plasma NF-κB1 and NF-κB2 concentration and thereby may activate nuclear factor kappa B which has a critical role to play in cancer development and progression.

In the context of these findings, it would be reasonable to conduct further studies on proteasome inhibitor application in the course of glial brain tumors. In this aspect the evaluation of proteasome activity would be very useful in clinical practice to assess a patient’s response to applied therapy.

## Supporting information

S1 Raw data (RAR)
